# Second Versus First Molar Extractions in Class II Division 1 Malocclusion Treatment: A Retrospective Longitudinal Outcome Study into Maxillary Canine, Premolar, and Molar Movement

**DOI:** 10.3390/jcm14010225

**Published:** 2025-01-03

**Authors:** Akkelien H. A. Oostenbrink, Ewald M. Bronkhorst, Johan W. Booij, Arjan J. A. Dieters, Yijin Ren, Anne Marie Kuijpers-Jagtman, Robin Bruggink

**Affiliations:** 1 Department of Orthodontics, University Medical Center Groningen, University of Groningen, Hanzeplein 1, 9713 GZ Groningen, The Netherlands; orthodontics@umcg.nl (A.H.A.O.);; 2 Department of Dentistry, Radboud Research Institute for Medical Innovation, Radboud University Medical Center, Philips van Leijdenlaan 25, 6525 EX Nijmegen, The Netherlands; 3 Private Practice in Gorinchem, 4207 AC Gorinchem, The Netherlands; 4 Center for Dentistry and Oral Hygiene, University Medical Center Groningen, University of Groningen, Hanzeplein 1, 9713 GZ Groningen, The Netherlands; 5 Department of Orthodontics and Dentofacial Orthopedics, School of Dental Medicine, Medical Faculty, University of Bern, Freiburgstrasse 7, CH-3010 Bern, Switzerland; 6 Faculty of Dentistry, Universitas Indonesia, Campus Salemba, Jalan Salemba Raya No. 4, Jakarta 10430, Indonesia; 7 Radboudumc 3D Lab, Radboud Institute for Health Sciences, Radboud University Medical Center, P. O. Box 9101, 6500 HB Nijmegen, The Netherlands; 8 Department of Dentistry, Section of Orthodontics and Craniofacial Biology, Radboud University Medical Center, Philips van Leijdenlaan 25, 6525 EX Nijmegen, The Netherlands

**Keywords:** orthodontics, longitudinal studies, malocclusion Angle Class II, 3-D imaging, maxillary first molar extraction, maxillary second molar extraction, treatment outcome

## Abstract

**Background/objectives:** This retrospective longitudinal outcome study comparing orthodontic extraction modalities, including extraction of maxillary first or second molars, aimed to compare the three-dimensional tooth movement of maxillary canines (C), premolars (P1, P2), and molars (M1, M2) in Class II division 1 malocclusion treatment with fixed appliances. **Methods:** A sample of 98 patients (mean age 13.20 ± 1.46 years) was selected for the M1 group, and 64 patients (mean age 13.20 ± 1.36 years) were chosen for the M2 group. Tooth movement was analyzed three-dimensionally on pre-treatment (T0) and post-treatment (T1) digital dental casts. Regression analyses compared the tooth movements (in mm) between the M1 and M2 groups. **Results:** The mean treatment duration for the M1 group was 2.51 ± 0.55 year, while, for the M2 group, it was 1.53 ± 0.37 year. The data showed limited distal movements of the C, P1, and P2 of approximately 2 mm in the M1 group and 1 mm in the M2 group during orthodontic treatment, but the M1 group exhibited significantly more distal movements than the M2 group (mean difference 1.11 to 1.24 mm). Vertical movements of the C, P1, and P2 in both groups were also minor (0.16 to 1.26 mm). The differences between groups did not exceed 0.2 mm and were not significant. Both treatment modalities resulted in a significant degree of anchorage loss with a distinct mesialization (8.40 ± 1.66 mm) of M2 in the M1 group and limited distalization (0.83 ± 0.98 mm) of M1 in the M2 group. **Conclusions:** The findings highlight the importance of thorough case evaluation when choosing between extraction modalities in Class II treatment. If a large distal movement of canines and premolars is required, additional anchorage mechanics should be considered.

## 1. Introduction

A Class II division 1 malocclusion, characterized by protrusion of the maxillary anterior teeth along with a Class II molar occlusion [1], poses an increased risk of trauma, which provides one of the main reasons to treat patients with this malocclusion [2]. Over time, several treatment options for this malocclusion have been established depending on factors such as patient age, growth pattern, facial soft tissue profile, crowding and proclination of the mandibular incisors, and patient compliance and preferences; additionally, the orthodontist’s experience and education play a role [3,4].

Among the various treatment modalities extractions are an acceptable option to correct a Class II division 1 malocclusion. Several variations in extraction pattern can be distinguished [5]. Extractions are typically applied in cases with moderate to severe crowding in both arches and/or with dental or dentoalveolar protrusion [6]. When crowding in the mandibular dental arch is mild and extractions seem excessive, a selective approach involving only maxillary extractions, such as the first or second premolars or molars, may be considered.

Maxillary premolar extractions offer a viable solution for addressing skeletal discrepancies through dental compensation [7]. This approach also addresses issues like upper anterior malalignment or pronounced teeth proclination when crowding in the mandibular arch is limited [8]. Moreover, achieving a Class II molar relationship is generally less demanding than achieving a Class I relationship, which depends on patient compliance for anchorage management; treatment-induced lower-incisor proclination is minimal [9]; and treatment duration is often shorter since obtaining a Class I molar relationship presents greater challenges. This strategy also induces soft tissue changes with an increase in the nasolabial angle but less retraction of the lower lip in two-premolar extraction cases [10].

Previous research has indicated that Class II division 1 malocclusion can also be effectively corrected using fixed appliances and maxillary first molar extractions [11,12]. However, closure of the extraction spaces resulted from significant mesial movement of the maxillary second molars, as measured cephalometrically, rather than the desired distal movement of the maxillary second premolars [13]. The latter also holds true when comparing maxillary first molar extractions and first premolar extractions [14].

Studies on maxillary second molar extraction in patients with Class II malocclusion have indicated that such extractions could be a preferred treatment strategy in cases of severe tooth damage, ectopic eruption or severe rotation of this molar, crowding in the posterior apical region, or excessive labial inclination of maxillary incisors without spacing. Research on maxillary first molar distalization after maxillary second molar extraction has shown a mean distal movement of the first molar of 1.2 mm (±SD 1.5 mm) compared to 0.0 mm (±SD 1.6 mm) in non-extraction cases [15]. Maxillary third molars must be in good shape, condition, and position when maxillary second molar extraction is considered. The preferred timing of extraction is when the third molar is approximately at the level of the cemento-enamel junction of the maxillary second molar [16,17]. Advantages of this approach include shorter treatment durations; easier distalization of first molars through the extraction site; less potential of reopening of the extraction space due to the third molar erupting in the remaining extraction area; and minor visibility if the space reopens [16,17,18]. Even if the patient turns out to be non-compliant, the situation does not worsen because the maxillary third molar will take its place in the dental arch. Paddenberg et al. [19] in 2023 give an overview of indications for maxillary first premolar or second molar extraction. They compared these two treatment options in a retrospective cohort study, showing that both treatment options are effective and that maxillary second molar extraction may have a positive effect on third molar alignment.

Several studies have evaluated treatment outcome for Class II division 1 malocclusion using different treatment modalities, applying cephalometric measurements and the Peer Assessment Rating (PAR) on dental casts [20,21]. While the latter is an assessment of the overall treatment outcome, it does not specifically address the amount of tooth movement. Measuring of tooth movement on digital models provides a more comprehensive understanding of the actual tooth movement in three dimensions by superimposing the digital dental casts [22,23,24,25,26]. A dental cast study on tooth movement after extraction of the maxillary first or second premolars in patients with fixed appliances using model superimposition showed that mesial movement of the first molars was comparable in both treatment modalities, at 4.7 mm (SD 1.6 mm) and 4.6 mm (SD 1.6 mm), respectively [26]. Longitudinal outcome studies on orthodontic treatment involving the extraction of maxillary first or second molars are scarce, and none of them reported on the three-dimensional displacement of maxillary canines and premolars [23,24,25].

Given the benefits of extracting the maxillary second molars, the drawbacks associated with extracting maxillary first molars, and the limitations in distal movement of the maxillary second premolars, this study aims to compare the three-dimensional movement of maxillary canines and posterior teeth after extractions of first and second molars in Angle Class II division 1 malocclusions, focusing on biomechanical efficiency and treatment time. The null hypothesis is that there is no difference in the extent of distal movement of maxillary canines and premolars between the M1 and M2 extraction groups.

## 2. Materials and Methods

### 2.1. Study Design

This is a retrospective longitudinal outcome study comparing digital dental casts of two groups of patients with a Class II division 1 malocclusion with different extractions in the maxilla who have been consecutively treated by a single, experienced orthodontist (J.W.B.). The outcome analysis was performed in an independent academic hospital. The reporting of this study followed the STROBE statement for observational research [27].

Treatment of patients was determined by the severity of the Class II malocclusion. In severe cases, where the disto-occlusion exceeded half the width of a premolar cusp, the first molars were extracted (M1 group). In milder cases, the second molars were extracted (M2 group). Patients were treated between December 1997 and September 2004 (M1 group) and October 2013 and December 2021 (M2 group). All patients were at the age of 10–15 years at the start of the treatment.

The inclusion criteria were a Class II division 1 malocclusion, fully erupted maxillary second molars, a well-formed mandibular dental arch, and maxillary third molars present and radiographically judged to have normal sizes and shapes.

### 2.2. Sample Size

The minimum sample size was calculated based on available research outcome data concerning treatment duration and the distalization of maxillary premolars [13,15]. A nomogram with 90% power at the 5% significance level was used requiring a minimal overall sample size of 84. To account for missing data, a minimal sample size of 50 in each group would be required.

All patient data were pseudo-anonymized prior to the analysis, and all participants had given informed consent. The study received approval from the Medical Ethical Committee (CMO) of the University Medical Center Groningen (METc 2020/460).

### 2.3. Treatment

#### 2.3.1. M1 Group (Extraction of Maxillary First Molars)

Treatment with fixed appliances started 2 weeks after extraction of the maxillary first permanent molars. Second maxillary molars were fully erupted before the extractions were carried out. All patients were treated with fixed appliances according to the principles of the light-wire technique. A detailed description of the treatment has been published earlier [28]. In short, at the start of treatment in the Class II correction phase, horizontal elastics (Light 5/16, T.P., Westville, IN, USA) were attached from a high-hat lock pin in the maxillary canine bracket to a ball end hook on the maxillary second molar band. The patient was instructed to replace these elastics once a week. Class II elastics (Medium 5/16, T.P., Westville, IN, USA) were used and had to be replaced every day. The wearing time was reduced as soon as a solid Class I premolar occlusion was reached. The second treatment phase consisted of establishing the correct torque of the maxillary anterior teeth, as well as the space closure and detailing.

After appliance removal, fixed retainers were bonded to all maxillary and mandibular anterior teeth. In cases of non-occlusion of the mandibular second molars, a buccal retention wire (0.195-inch Wildcat, GAC, Central Islip, NY, USA) was bonded, connecting the first and second mandibular molar to keep these teeth in position. These buccal retention wires were removed after the complete eruption of the maxillary third molars.

#### 2.3.2. M2 Group (Extraction of Maxillary Second Molars)

Treatment was performed according to the same two stages, as described for the M1 extraction group. The first phase focused on correcting the Class II occlusion of the first molars. After placement of fixed appliances, without bracketing the maxillary premolars, and extraction of the maxillary second molars, jigs (Medium 3/16, T.P., Westville, IN, USA) were used to distalize the maxillary first molars. After sufficient distalization of the maxillary first molars, the jigs were removed and Class II elastics were applied to retract the maxillary anterior teeth. At this point, the maxillary premolars were included in the fixed appliances, still using the same 0.16″ wire. The maxillary premolars shifted distally spontaneously following distalization of the maxillary first molars. During the second phase, the 0.16″ wire was replaced with an 0.18″ wire combined with an 0.14″ torquing auxiliary. Residual diastemas were closed and the correct torque and position of the teeth was established, sometimes still with limited use of the Class II elastics. After treatment, the retention procedure was the same as in the M1 group.

### 2.4. Data Collection

For each patient, dental casts were collected before the start of the treatment (T0) and after treatment (T1). The dental casts were digitized using a 3Shape D710^®^ Dental Laser scanner (3Shape, Copenhagen, Denmark). The resulting digital models were exported as Standard Tessellation Language (STL) files.

The models were imported into 3Shape OrthoAnalyzer™ software (v1.9.3.0, 3Shape^®^, Copenhagen, Denmark). The occlusal plane and midsagittal plane were determined in an OrthoAnalyzer to align the models in a standardized position. Subsequently, the segmentation module was used automatically to separate the individual teeth from the total model. The resulting model and teeth were exported again as separate STL files.

### 2.5. Variables

#### Three-Dimensional Tooth Movement

**Figure 1:**
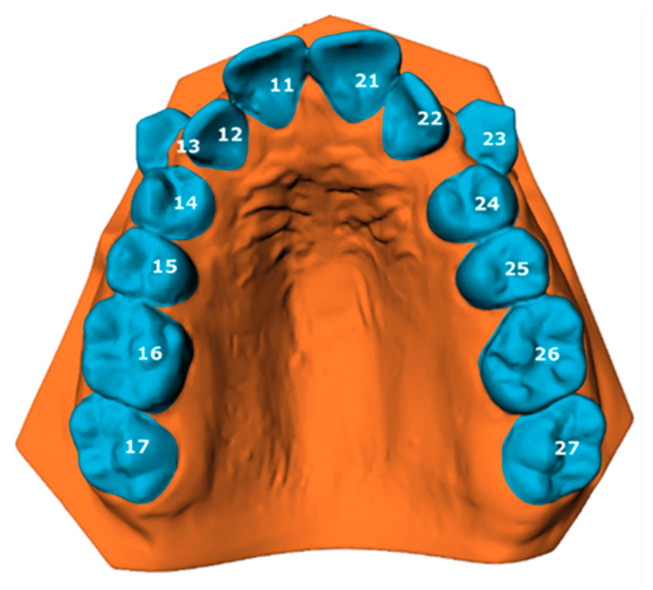
Loaded T0 model with its segmented teeth visualized. Each tooth is numbered according to the Féderation Dentaire International (FDI) teeth numbering.

**Figure 2:**
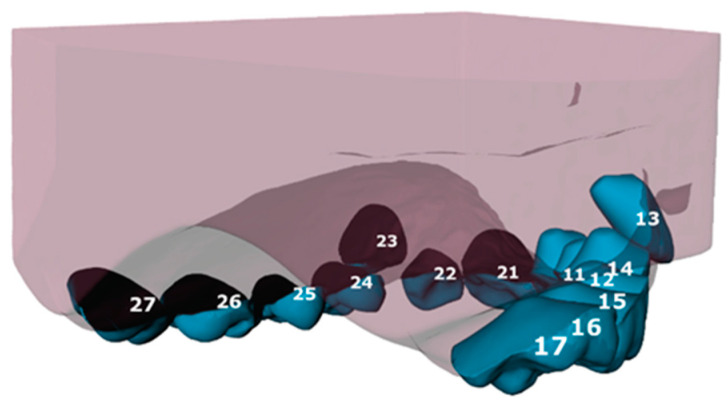
Teeth with their roots still attached (tooth 11–17) in comparison with teeth with their roots cut off (tooth 21–27). Each tooth is numbered according to the Féderation Dentaire International (FDI) teeth numbering.

**Figure 3:**
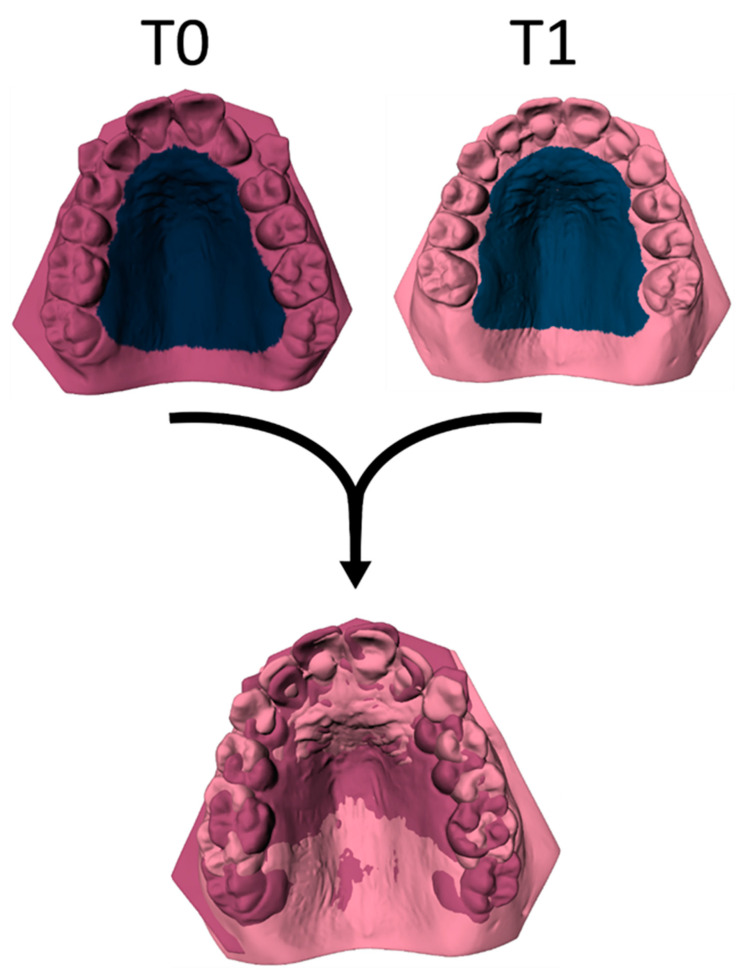
The T1 model is superimposed over the T0 model by using an ICP algorithm with the palate as the reference area.

**Figure 4:**
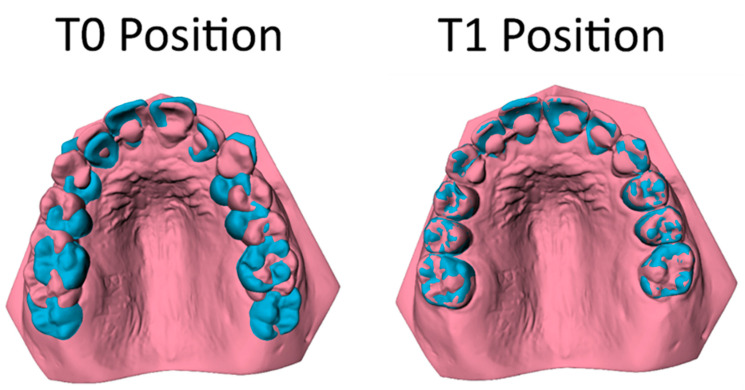
The superimposition of the teeth (pink) on their T0 location (blue) (**left picture**) towards the T1 maxillary model (**right picture**).

**Figure 5:**
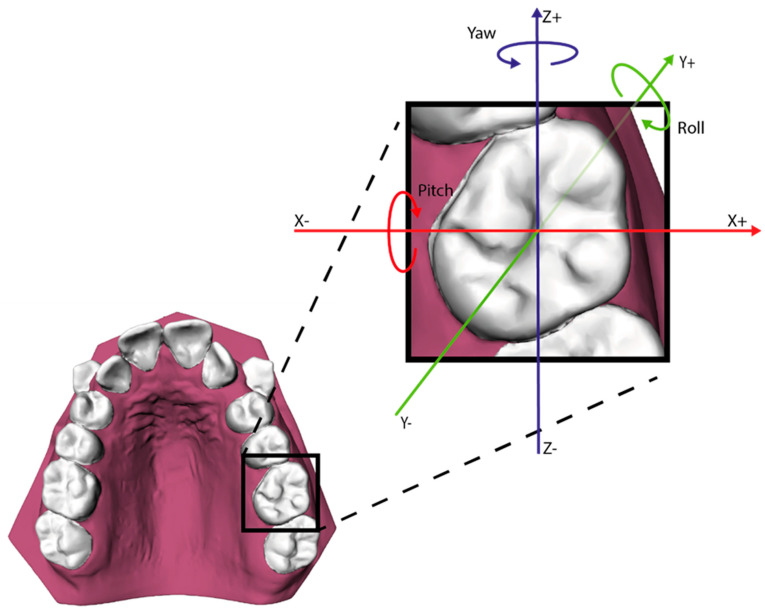
The six degrees of freedom for describing the movement of each tooth.

The tooth movement between T0 and T1 was evaluated using the ‘Tooth Movement Analyzer’ module in 3DMedX^®^ (v1.2.32.0, 3D Lab Radboudumc, Nijmegen, The Netherlands). This process, displayed by Figure 1, Figure 2, Figure 3, Figure 4 and Figure 5, has the following steps. First, the T0 maxillary model with its corresponding segmented teeth was loaded into the software. The corresponding Féderation Dentaire International (FDI) teeth numbering was displayed over each tooth to check if the segmentation algorithm had labeled all teeth correctly (Figure 1). Second, the roots of the teeth were automatically indicated and removed from the models, as they will intervene with the next superimposition steps (Figure 2). Third, the T1 maxillary model was loaded and superimposed on the T0 model using an iterative closest point (ICP) algorithm with the palatal surface as reference (Figure 3).

As both maxillary models are now aligned, the mutual differences between corresponding teeth can be calculated by superimposing the teeth on T0 towards the T1 maxillary model (Figure 4). The movement of each individual tooth is expressed in six degrees of freedom; the translation in millimeters for the x-, y-, and z-axis and the rotation in degrees for the pitch, roll, and yaw (Figure 5). Positive values on the z-axis depict the mesial movement of the teeth while negative values represent their distal movement. This study focuses on the distalization of the maxillary cuspids (C), first premolar (P1) and second premolar (P2); thus, distalization of these teeth is displayed by a negative value on the z-axis.

To assess the intra-observer reliability of the procedure, superimposing of the scans was conducted twice for 20 models by the same observer (A.O.). To assess the interobserver reliability, superimposing of the scans was performed by a second observer for 20 models (A.D.)

### 2.6. Statistical Analysis

The statistical analysis was performed using R, version 3.6.1 (R statistical computing and graphics www.r-project.org, accessed on 13 December 2023). Outcomes for the amount of movement of the maxillary C, P1 and P2 in the x, y, and z direction, as well as the Euclidean distances, are presented as a variable with a mean ± SD and a range. Within each group, the mean increments and SD (T1–T0) were calculated for the movement of these teeth. To compare the amount of tooth movement between the two groups (M1 and M2) a multilevel regression analysis with a random intercept for each patient was applied. The amount of tooth movement in the M1 group was deducted from the amount of tooth movement in the M2 group. If the M2 group had a higher value than the M1 group, this would result in a positive estimate value. All residuals were visually inspected to check for potential outliers or influential points; none were encountered. Statistical significance was set at a *p*-value < 0.05.

For the 3D measurements, intra-and interobserver reliability was determined by computing the Pearson’s correlation coefficients between the two measurements. To detect any systematic differences between the measurements, paired sample *t*-tests were employed. The duplicate measurement error (DME, random error) was quantified by dividing the standard deviation of the difference between the two observations by √2.

## 3. Results

### 3.1. Sample Descriptives

A total of 162 patients were included (Table 1). Among these, 98 patients were categorized into the M1 extraction group, with a mean age of 13.20 ± 1.46 years, comprising 53 males and 45 females. The M2 extraction group consisted of 64 patients, with a mean age of 13.20 ± 1.36 years, including 25 males and 39 females. The mean treatment duration in the M1 group was 2.51 ± 0.55 year, and it was 1.53 ± 0.37 year in the mean group.

For every tooth type, left and right teeth were combined. If a tooth was clinically absent at the start of treatment (T0), this tooth was excluded from the analysis. Consequently, in the M1 extraction group, there were 182 maxillary canines, 196 maxillary first premolars, 176 maxillary second premolars, and 187 maxillary second molars. In the M2 extraction group, 120 maxillary canines, 128 maxillary first premolars, 116 maxillary second premolars, and 128 maxillary first molars were included.

### 3.2. Reliability of the Method

The results of the intra- and interobserver reliability tests for the 3D measurements are shown in Table 2. Significant differences were found for the mean difference for all interobserver comparisons and the Euclidean distance for the intra-observer comparison. However, differences were small (−0.13 to 0.13 mm). The interobserver reliability, as expressed by the Pearson’s correlation coefficient, ranged from 0.94 to 0.98, and the intra-observer reliability ranged from 0.99 to 1.00.

### 3.3. Tooth Movement

**Figure 6:**
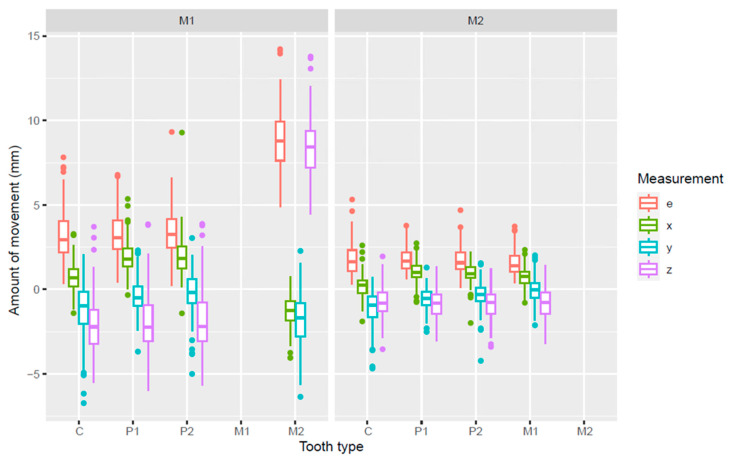
Box plot of the amount of movement (mm) in three dimensions of the maxillary C, P1, P2, and M2 in the M1 extraction group and of the maxillary C, P1, P2, and M1 in the M2 extraction group. e = Euclidean distance; x, y, z = Movement on the x-, y-, z-axis, respectively.

In Table 3 and Figure 6, the mean increments of the maxillary C, P1 and P2 in the M1 group are compared with those of the M2 group. As was shown in Figure 5, positive values on the z-axis depict mesial movement of the teeth, while negative values represent their distal movement.

The maxillary C, P1, and P2 in the M1 extraction group showed more distal movement (z-axis) than the same teeth in the M2 extraction group. The mean increments for transversal movements (x-axis) and Euclidian distances were also larger in the M1 group.

### 3.4. Three-Dimensional Measurements Analysis

In Table 4, the increments of the maxillary C, P1, and P2 of the M1 group are compared with those of the M2 group. A positive estimate value for the z-axis means a more distal movement for those teeth in the M1 group than in the M2 group. For all teeth listed in Table 4 (C, P1 and P2), distal movement in the M1 group was significantly larger than in the M2 group, with a mean difference of 1.11 to 1.24 mm for the z-axis.

## 4. Discussion

This study aimed to investigate the extent of tooth movement of the maxillary canines and premolars after extracting the maxillary second molars compared to extraction of the maxillary first molars in treating a Class II division 1 malocclusion.

The null hypothesis was that there is no difference in the extent of distal movement of these teeth between the M1 and M2 extraction groups. This hypothesis was rejected, as the findings show that the maxillary C, P1 and P2 in the M1 extraction group were moved more distally than in the M2 extraction group. When the first molars were extracted, the maxillary C, P1, and P2 moved about 2 mm distally (Table 3), while, in the M2 group, the distal movement was limited to less than 1 mm, which was a significant difference.

In our study, we used a contemporary method to analyze tooth movement after maxillary first or second molar extraction three-dimensionally; however, unfortunately, we only can compare the findings to cephalometric studies. In an earlier cephalometric study on the same patient group with a Class II malocclusion using Pancherz’s Sagittal Occlusion (SO) analysis, it was also found that space closure after extraction of the maxillary first molars was mainly achieved with a mesial movement of the second molars of 9.9 mm rather than a distal movement of the P2, which was limited to 1.4 mm [13,29]. Although distal movement of the maxillary canines and premolars was significantly different between the two extractions groups, the clinical relevance of this difference is minor. The most important finding is that both treatment modalities resulted in a significant degree of anchorage loss, with a distinct mesialization (8.40 ± 1.66 mm) of the maxillary second molars in the M1 extraction group and limited distalization (−0.83 ± 0.98 mm) of the maxillary first molars in the M2 extraction group. This complies with the spontaneous space closure that can be expected after permanent first molar extraction [30]. It also demonstrates that treatment mechanics to preserve anchorage should be carefully employed. If a large distal movement of the canines and premolars is required, additional intra-oral anchorage mechanics supported by temporary anchorage devices should be considered [31,32,33].

Apart from the sagittal movement of the teeth, transversal (x-axis), coronal (y-axis) and 3D movement (e) were measured in this study. Comparisons of the intrusion and extrusion movement (y-axis) between all tooth types for the M1 and M2 group were found to be non-significant. Despite the fact that some teeth had not fully erupted at T0, little movement was found on the y-axis, with small differences occurring between the two groups. This means that extraction of the M1 or the M2 had a comparable and minor effect for vertical movements.

Although significant, the differences for the Euclidean distance and transversal movement were also small between the M1 and M2 extraction group (Table 4). With differences for movement on the x- and z-axis being significant, differences in the Euclidean distances for those tooth types can be expected to be significant as well, as they are highly correlated. Transversal development (x-axis) in the maxillary arch is a well known aspect of orthodontic treatment of Class II division 1 malocclusions. Due to the more distal position of the mandibular molars in relation to the maxillary molars in a Class II malocclusion, the maxillary premolar region adapts to the smaller mandibular anterior region. When correcting a Class II malocclusion, transversal buccal development of the maxillary dental arch is often necessary [34]. The difference in buccal transversal movement shows that this movement was larger for the M1 extraction group, although the difference was less than 1 mm.

As tooth movement occurs along the dental arch, the chosen axes in this study give a good indication for the amount of movement but are not completely correct for all tooth types. Considering the shape of the dental arch, distal movement for a maxillary molar corresponds more to the sagittal plane than distal movement for a maxillary cuspid. For future research, we propose that every tooth should have its own coordinate system. The development of such a tooth-specific coordinate system may give a more accurate representation of the movement of the separate teeth. This could be solved by aligning these tooth-specific coordinate systems to the dental arch based on the axis defining distalization and mesialization.

Our study included 98 participants in the M1 group and 64 in the M2 group, exceeding the initially required sample size of 50 per group. This larger sample size was achieved by including all available consecutive cases. Both groups were treated in a private practice by the same orthodontist using the light-wire technique, utilizing standardized brackets featuring a vertical slot and the same type of Australian wires that have been used in orthodontics for over half a century. Besides the increased operator experience, differing time intervals are not expected to have influenced the comparative results.

Apart from studies from our research group, there are very few other longitudinal outcome studies on orthodontic treatment involving the extraction of maxillary first or second molars, and none have reported on the displacement of maxillary canines and premolars [15,16,17]. The increased sample size also accounts for patient variability and offsets potential data gaps, which are common in longitudinal studies like ours, where the development of the dentition also plays a role. The mean age at T0 coincided with the end of the eruption phase of the permanent teeth to be measured. In the end, it was revealed that all maxillary first premolars were present in both groups; however, some second premolars and canines were absent at T0, and hence their displacement during treatment could not be measured.

The intra- and interobserver reliability of the 3D measurements in this study was high: 0.94–1.00, as established by the Pearson correlation coefficient (Table 2). The interobserver mean differences showed statistical significance but the differences were very small (0.04–0.13 mm) and clinically irrelevant. Nevertheless, the 3D measurements used in this study give a better depiction of the displacement of the teeth than a depiction given by 2D cephalometric measurements. In 2D imaging, tooth rotation may be misinterpreted as mesialization or distalization, which happen in the same sagittal plan. Also, projection enlargement can be a cause for misleading measurements. Lastly, taking measurements on a 2D depiction of a 3D object is highly dependent on the angle at which the 2D image is reconstructed. This can also cause a smaller or larger depiction of the part that is to be measured.

When faced with the choice of maxillary molar extraction, both first and second maxillary molar extractions can result in a dentition with a non-extraction “eight-premolar” appearance, which may enhance dentofacial esthetics. Second molar extraction may be a preferable option for patients and parents, as it minimizes visible gaps and helps in maintaining a largely continuous dental arch during treatment. In contrast, first molar extraction creates a noticeable diastema that persists for much of the treatment period. Furthermore, closed extraction spaces often tend to reopen slightly after treatment. In the case of maxillary premolar or maxillary first molar extraction, such gaps are more visible than with maxillary second molar extraction. In cases where first molars have a poorer prognosis, such as those affected by Molar–Incisor Hypomineralization (MIH), the decision to extract is often more straightforward. The role of the general dentist is also important, as they should be well informed about the different treatment options to provide appropriate guidance and support to parents and patients.

A notable finding is the shorter treatment duration associated with second molar extractions, averaging 1.53 ± 0.37 years compared to 2.51 ± 0.55 years for first molar extractions. This difference can be partially attributed to variations in the initial severity of malocclusions. Nonetheless, the treatment times for second molar extractions in this study are shorter than the previously reported average of 30.1 months for adolescent orthodontic treatment in general [35], and they are also shorter than the 24.00 ± 5.90 months observed by Paddenberg et al. [19] following maxillary second molar extractions. Unlike prior research that utilized cervical pull headgear (CPHG) for anchorage purposes, this study employed distalization jigs, which may have contributed to the reduced treatment times due to their decreased reliance on patient compliance.

The limitations of this study, as discussed before, include not using standardized 3D axes and the study design. Additionally, the study was based on data collected in a single-center and single-practitioner setting. The lack of diversity in practitioners and treatment settings limits the generalizability of the results. Moreover, the retrospective nature of the study introduces inherent biases and limitations. The disparity in malocclusion severity between the groups treated with maxillary M1 extraction and those treated with maxillary M2 extraction adds to this limitation. A prospective approach would offer more controlled and rigorous evaluation of the treatment modalities in question. However, obtaining ethical permission for a prospective study involving extractions can be challenging. This emphasizes the need for gathering as much information as possible from retrospective data to broaden our understanding of orthodontic treatment modalities.

## 5. Conclusions

Orthodontic treatment of a Class II division 1 malocclusion with fixed appliances and involving either maxillary M1 or M2 extractions is associated with a significant degree of anchorage loss, particularly in cases where M2 is extracted. While M1 extractions also result in anchorage loss, the degree is notably lower. These findings highlight the importance of thorough case evaluation when determining the appropriate extraction strategy in Class II treatment. In cases requiring a substantial distal movement of canines and premolars, the use of additional anchorage mechanics should be considered to optimize treatment outcomes.

## Figures and Tables

**Table 1:** Group characteristics. Characteristics of the M1 and M2 extraction groups (means, standard deviations and range). Age at T0, age at T1, and treatment duration.

Parameter	Groups					
	M1 Extraction (*n* = 98)			M2 Extraction (*n* = 64)		
	Mean	SD	Range	Mean	SD	Range
Age T0 (years)	13.20	1.46	10.50–17.20	13.20	1.36	10.30–16.30
Age T1 (years)	15.70	1.58	12.40–19.80	14.70	1.45	11.30–18.10
Treatment duration (years)	2.51	0.55	1.36–4.49	1.53	0.37	0.82–2.64

T0 = start of treatment; T1 = end of active treatment.

**Table 2:** Intra- and interobserver reliability for 3D measurements. The random error is shown by the duplicate measurements error (DME). Mean differences are tested with a paired samples *t*-test. The reliability was calculated using a Pearson correlation coefficient (PCC).

		DME	PCC	Mean Diff (mm)	95% CI (mm)	*p*
**Inter**	e	0.44	0.96	−0.13	−0.21; −0.05	**0.002**
	x	0.21	0.98	0.04	0.00; 0.08	**0.049**
	y	0.33	0.94	0.12	0.06; 0.18	**<0.001**
	z	0.69	0.94	0.13	0.01; 0.26	**0.038**
Intra	e	0.08	1.00	−0.02	−0.04; −0.00	**0.019**
	x	0.05	1.00	0.00	−0.01; 0.01	0.637
	y	0.13	0.99	0.01	−0.01; 0.04	0.246
	z	0.06	1.00	0.01	−0.00; 0.02	0.181

The statistical significant *p*-values in bold.

**Table 3:** Three-dimensional measurements. Mean movement increments of the maxillary canines (C), first premolars (P1), second premolars (P2), first molars (M1) and second molars (M2). Results are grouped for the M1 extraction group and the M2 extraction group. The Euclidian distance (e) is calculated from the movement on the x-, y- and z-axis.

		M1 Extraction Group			M2 Extraction Group		
Tooth Type	Axis	Mean (mm)	SD (mm)	[Min; Max] (mm)	Mean (mm)	SD (mm)	[Min; Max] (mm)
C	e	3.17	1.42	0.35; 7.82	1.82	1.03	0.27; 5.32
	x	0.73	0.88	−1.41; 3.28	0.19	0.70	−1.90; 2.60
	y	−1.26	1.60	−6.72; 2.07	−1.13	1.08	−4.68; 0.72
	z	−2.09	1.52	−5.51; 3.71	−0.75	0.91	−3.54; 1.95
P1	e	3.25	1.22	0.41; 6.78	1.76	0.70	0.61; 3.77
	x	1.89	0.86	−0.33; 5.36	1.01	0.63	−0.75; 2.73
	y	−0.41	0.98	−3.68; 2.31	−0.56	0.62	−2.51; 1.29
	z	−2.01	1.61	−5.99; 3.86	−0.83	0.90	−3.07; 1.37
P2	e	3.33	1.28	0.20; 9.32	1.75	0.75	0.11; 4.69
	x	1.91	1.05	−1.42; 9.28	0.94	0.62	−1.99; 2.21
	y	−0.16	1.20	−5.00; 3.04	−0.37	0.80	−4.23; 1.55
	z	−1.95	1.66	−5.69; 3.88	−0.83	0.95	−3.40; 1.26
M1	e	-	-	-	1.59	0.75	0.38; 3.73
	x	-	-	-	0.72	0.61	−0.79; 2.34
	y	-	-	-	−0.03	0.74	−2.12; 2.01
	z	-	-	-	−0.83	0.98	−3.24; 1.44
M2	e	8.85	1.93	4.89; 14.22	-	-	-
	x	−1.30	0.91	−4.05; 0.75	-	-	-
	y	−1.77	1.51	−6.35; 2.28	-	-	-
	z	8.40	1.66	4.45; 13.78	-	-	-

**Table 4:** Analysis of the 3D movements of the maxillary C, P1, and P2 in the M1 group vs. the M2 group difference in increments, M2 minus M1, were tested with a multilevel regression analysis.

Tooth Type	Axis	Estimate (mm)	95% CI (mm)	*p*
C	e	−1.30	−1.66; −0.93	**<0.001**
	x	−0.54	−0.74; −0.33	**<0.001**
	y	0.11	−0.29; 0.51	0.587
	z	1.24	0.86; 1.61	**<0.001**
P1	e	−1.48	−1.74; −1.21	**<0.001**
	x	−0.87	−1.08; −0.67	**<0.001**
	y	−0.15	−0.36; 0.07	0.196
	z	1.14	0.77; 1.52	**<0.001**
P2	e	−1.58	−1.87; −1.30	**<0.001**
	x	−0.93	−1.18; −0.57	**<0.001**
	y	−0.20	−0.50; 0.10	0.201
	z	1.11	0.70; 1.51	**<0.001**

The statistical significant *p*-values in bold.

## Data Availability

The data presented in this study are available on request from the corresponding author. The data are not publicly available due to privacy limitations.
